# Acoustophoretic Characterization and Separation of Blood Cells in Acoustic Impedance Gradients

**DOI:** 10.1103/PhysRevApplied.20.024066

**Published:** 2023-08-25

**Authors:** Mahdi Rezayati Charan, Per Augustsson

**Affiliations:** Department of Biomedical Engineering, Lund University, Ole Römers Väg 3, 22363 Lund, Sweden

## Abstract

Single-cell phenotyping based on biophysical properties is a promising tool to distinguish cell types and their response to a given condition, and charting such properties also enables optimization of cell separations. Isoacoustic focusing, where cells migrate to their points of zero acoustic contrast in an acoustic impedance gradient, added the effective acoustic impedance of cells to the directory of biophysical properties that can be utilized to categorize or separate cells. This study investigates isoacoustic focusing in a stop-flow regime and shows how cells migrate towards their isoacoustic point. We introduce a numerical model that we use to estimate the acoustic energy density in acoustic impedance gradient media by tracking particles of known properties, and we investigate the effect of acoustic streaming. From the measured trajectories of cells combined with fluorescence intensity images of the slowly diffusing gradient, we read out the effective acoustic impedance of neutrophils and K562 cancer cells. Finally, we propose suitable acoustic impedance gradients that lead to a high degree separation of neutrophils and K562 cells in a continuous-flow configuration.

## I Introduction

Measuring cells’ inherent biophysical properties at the single-cell level is commonly used in microfluidics to classify cells based on their unique biophysical signature [1,2]. The high precision and flexibility for single-cell phenotyping offered in microfluidic systems have established fast and automated routines for the separation of rare cell populations, such as white blood cell (WBC) subpopulations [3] and cancer cells [4]. Different biophysical properties, including size [5,6], density [7], deformability [8], electrical impedance [9], magnetic [10,11], and optical [12] properties, have been utilized to analyze various types of cells for cytometry applications and to improve the separation outcome of target cells in a label-free format [13]. Moreover, several studies have observed altered “biophysical fingerprints” due to different conditions such as disease [14,15], cell growth cycle [16], activation level [17], and apoptosis [18]. Within the microfluidic toolbox, the present work deals with acoustofluidics to measure the effective acoustic impedance (*Z*_*c*_) at the single-cell level and to discriminate cells by this property.

The motion of micro-objects, such as cells or microparticles, in a microchannel hosting a standing acoustic wave is mainly governed by the acoustic radiation force, providing a controlled lateral displacement of micro-objects along the gradient of the acoustic potential. Cell and particle migration in the acoustic field depends on their size and differences in mass density and compressibility relative to the surrounding medium [19,20]. This technique has been extensively implemented in various applications, including droplet microfluidics [21,22], microparticle and cell washing [23], trapping and concentration of exosomes and microvesicles [24], and, importantly, for separation purposes [25–27]. Acoustophoretic separation is contact-free, and the driving acoustic radiation force is comparable in magnitude to that of centrifugation, with the clear benefit of being straightforwardly implemented for inline processing.

Most studies on the acoustophoretic separation of cells utilize an approach where cells are suspended in a homogeneous medium and compete toward the acoustic nodal position due to their positive acoustic contrast and size [28,29]. A limiting factor in this regime is the strong size dependency in acoustophoresis combined with the fact that subgroups of cells often have overlapping size distributions [30]. The strong size dependency can be overcome by altering the suspending medium’s acoustic properties such that different cells in a mixture can have positive, zero, or negative acoustic contrast [31–33]. However, in such a configuration, all cells will have very low acoustic contrasts, and thus their motion becomes susceptible to acoustic streaming. Consequently, for cells of similar but nonoverlapping acoustic properties, deterministic size-insensitive separation is not feasible in homogeneous media [34].

Although cell and particle motion in the homogenous configuration have been well studied, the acoustophoretic motion of cells suspended in inhomogeneous fluids lacks a detailed analysis of their motion based on the governing parameters since its recent introduction to the acoustofluidics field. It was discovered that at the interface between two coflowing aqueous fluids of different densities and compressibility, an acoustic body force arises due to the gradients in the material properties of the fluid, which leads to fluid relocation or stabilization [35,36], and the suppression of acoustic streaming [37–39]. Based on the observed phenomenon, a size-insensitive method called isoacoustic focusing (IAF) was developed to analyze cells based on their effective acoustic impedance [40]. In this method, cells suspended in an acoustofluidic glass-silicon chip migrate sideways in a fluid with a smooth acoustic impedance (*Z*_*m*_) profile toward an equilibrium position known as the isoacoustic point (IAP), where the acoustic contrast (Φ) between the cell and the surrounding liquid is zero, and the sideways displacement due to the radiation force ceases. For cells at rest in their IAP, it has been established that the acoustic impedance of the cell (*Z*_*c*_) ≈ *Z*_*m*_ such that *Z*_*c*_ can be approximated by measuring the *Z*_*m*_ of the fluid surrounding the cell. IAF was later adopted to separate cancer cell line cells with higher or lower acoustic impedance than peripheral blood mononuclear cells by tuning the acoustic impedance of the liquids across the channel [41].

This study aims to investigate the cell motion patterns in IAF during stopped flow to better understand under what conditions individual cells reach their IAP and how different parameters, including cell radius (*a*), density (*ρ*_*c*_), compressibility (*κ*_*c*_), acoustic energy density (*E*_ac_), and the gradient in material properties in the fluid, impact the cell trajectories. In that regard, stopping the flow provides a better ground over a continuous-flow format to monitor single cells’ trajectories over time and show that cells can stay in their IAP for some time while the acoustic impedance gradient continuously evolves. We set up a numerical model to simulate trajectories of cells and particles in acoustic impedance gradient media, and we experimentally determine *E*_ac_ in inhomogeneous media by fitting the model to trajectories of microparticles of known properties. We show that the common approach of approximating *E*_ac_ for a stratified medium based on tracking microparticles in a corresponding homogenous system can lead to large errors. We then study the suppression of acoustic streaming, and we track cells as they approach their IAP and measure *Z*_*c*_, and compare it to simulations. In our previous study [34], we concluded that acoustic streaming in homogeneous media limits deterministic cell separation; however, in the present work, we explain that nonexisting acoustic streaming leads to a better resolution in cell separation. Finally, we demonstrate the separation of a mixture of cells in a gradient of diffusing iodixanol in a continuous-flow format.

## II Materials and Methods

### A Cell culture and staining

The experiments were conducted on human neutrophils and K562 cells (from Pereira lab, Division of Molecular Medicine and Gene Therapy, Lund University). Neutrophils were obtained from blood samples of healthy volunteers using an EasySep™ Direct Human Neutrophil Isolation Kit (STEMCELL Technologies, Norway). Blood sample collection was done with written informed consent and was approved by the Swedish ethical review authority (ref. no. 2020-05818) in line with the Helsinki Declaration. Isolated neutrophils were centrifuged, washed, and resuspended in phosphate-buffered saline (PBS, Sigma-Aldrich). K562 cells were cultured in T75 flasks with RPMI-1640 medium (Sigma-Aldrich) supplemented with 10% fetal bovine serum (FBS, Sigma-Aldrich) and 1% penicillin-streptomycin (Sigma-Aldrich). The cells were maintained at 37 °C in a 5% CO_2_ atmosphere and were passaged every 2–3 days.

For microscopy imaging, CellTracker™ Green CMFDA (ThermoFisher) and CellTracker™ Red CMTPX (ThermoFisher) were used as fluorescent dyes. A required number of cells were collected in a tube and centrifuged. The supernatant was discarded, and the cells were incubated at 37 °C for 20 min in PBS with 1 μM of fluorescence dye. Finally, the cells were resuspended in PBS with 2% FBS at a concentration of 2 × 10^6^ cells per ml after undergoing two more centrifugation steps to wash the remaining dye in the medium.

### B Experimental setup

**FIG. 1:**
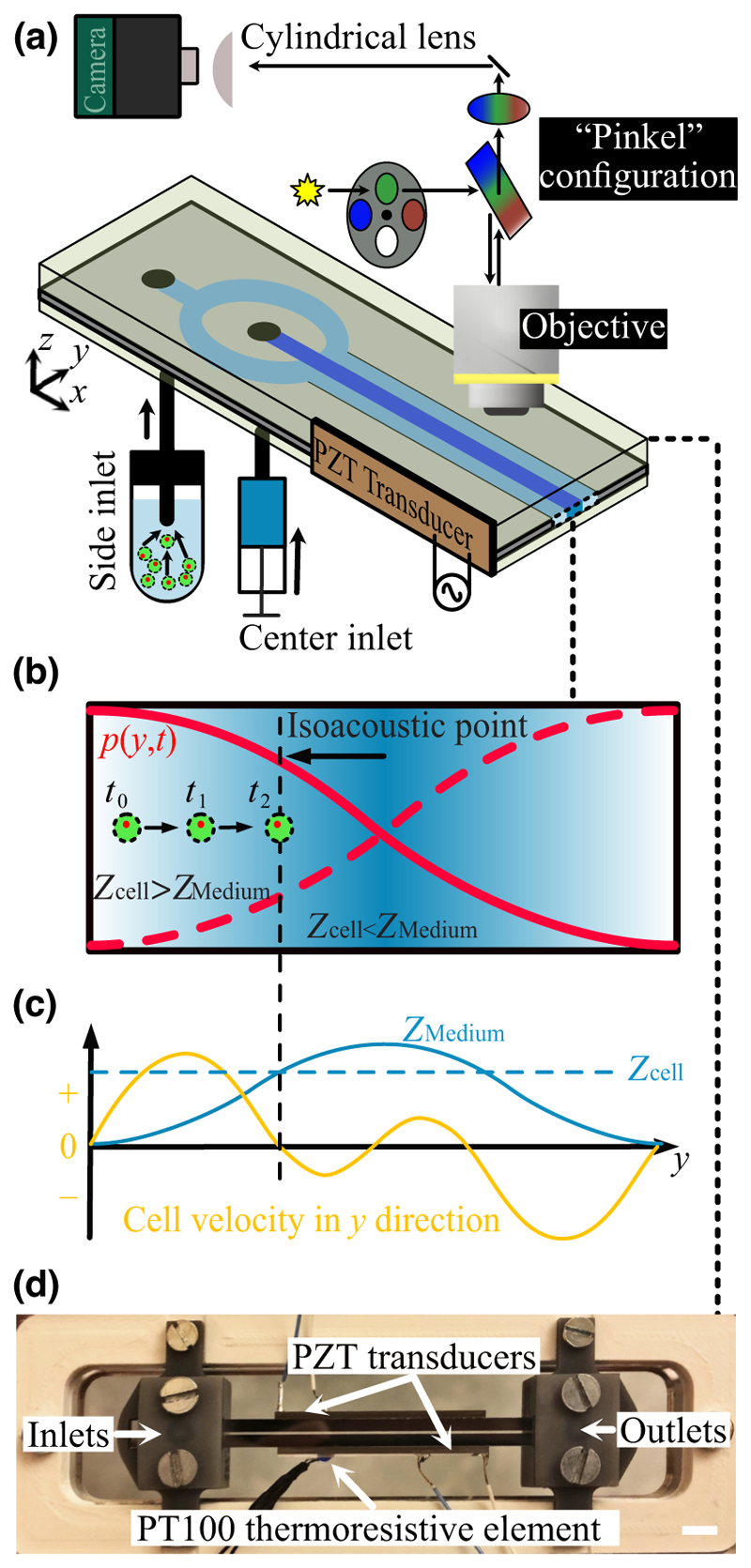
Experimental setup showing (a) an illustration of the isoacoustic focusing (IAF) chip and the optical and flow configuration, (b) a schematic of the IAF principle where cells (green) are pushed by an acoustic resonant pressure field *p* (red curves) towards their IAP. (c) Plot of the velocity of a cell as it reaches the IAP. (d) Photograph of the chip placed in its holder. Scale bar 5 mm. (b),(c) are reproduced from Ref. [40] with permission.

An acoustofluidic glass-silicon-glass chip was fabricated to have a length of 70 mm and a width of 4 mm (GeSim Bioinstruments and Microfluidics mbH, Germany). The designed channel of width *W =* 375 μm was etched through an *H =* 150 μm thick silicon wafer by deep reactive ion etching and was anodically sandwiched between two glass lids [Fig. 1(a)]. A piezoceramic transducer (Pz26, Ferroperm™ piezoceramics, Meggit A/S, Denmark) resonant at 2 MHz and 30 × 2.0 × 1.0 mm^3^ in size was bonded to the side of the chip using cyanoacrylate glue (Loctite Super Glue, Henkel Norden AB, Stockholm, Sweden). The transducer was driven by a function generator (AFG3022B, Tektronix, Inc., Beaverton, Oregon, USA) to deliver a frequency sweep (1.85–1.95 MHz), with 1 ms sweep time and different applied voltages as measured over the piezo with an oscilloscope (TDS1002, Tektronix, Inc., Beaverton, Oregon, USA). The resonant frequency was determined by observing the focusing of particles (1.90 MHz), and the frequency sweep was used to make the acoustic field as uniform as possible along the imaging field [42]. A Pt100 thermoresistive element was glued to the transducer to monitor that the chip temperature was always at room temperature (approximately 22 °C).

Images were recorded using an epifluorescence microscope (BX51WI, Olympus Corporation, Tokyo, Japan) with a 10×, 0.3 NA objective and a CMOS camera (ORCA-Flash 4.0 V3, Hamamatsu Photonics KK, Japan). The periodicity of all imaging was 100 ms per frame throughout the work. When imaging in two fluorescence channels, the repetition rate of both channels was 10 Hz but with an offset of 30 ms. The exposure time for all the fluorescence channels was 20 ms throughout the experiments. A triple-wavelength LED unit (CoolLED pE-300^ultra^, UK) was used for the illumination of fluorescent polystyrene (PS) particles with nominal diameters of 1.1 and 4.9 μm (Molecular Probes, Thermo Fisher Scientific, Waltham, MA), stained cells, and the suspension media containing a fluorescent tracer molecule. The LED unit includes three different single-band excitation filters (ET395/25x for fluorescent dextran tracer molecules, ET480/30x for PS particles, and ET560/25x for cells, Chroma Technology Corp, USA). In the microscope filter turret, a required multiband emission filter (69013bs, Chroma Technology Corp, USA) with the corresponding dichroic mirror (69013m, Chroma Technology Corp, USA) was mounted to establish the Pinkel configuration [Fig. 1(a)]. The Pinkel set allows us to perform a fast sequential multichannel acquisition. The excitation channels were activated through triggering from multiple digital inputs. The exact timing of the acquisition was controlled via trigger signals from an Analog Discovery 2 data acquisition system (Digilent, USA). Synchronized signals triggered the CMOS camera and different LED unit excitation channels for imaging. A cylindrical lens with a focal length of 300 mm was inserted into the light path to acquire elliptically shaped defocused images of particles and cells. The cylindrical lens was located between the camera and the objective lens at a distance of 20 mm from the camera sensor.

At the main channel inlet, three streams join in a trifurcation in which the two side streams are routed via a two-unit syringe pump (Tricontinent C-series, Gardner Denver, USA), while the center stream is regulated by using a pressurized tube holder (Elveflow adaptor for Falcon© tubes). A motorized four-port, two-way diagonal valve controls the inlet flow streams to facilitate the quick stop of the flow in the channel by a short circuit. The detailed procedure with the schematic is explained in Ref. [38]. Experiments were carried out in a bubble-free system to guarantee a quick stop of the flow compared to the timescale of the experiments and to avoid a drift in the flow along *x*.

### C Operating principle

IAF analyses cells’ sideways migration in a half-wavelength resonant acoustic pressure field *p*(*y, t*) and reports their effective acoustic impedance (*Z*_*c*_). In this technique, cells are suspended in a microchannel filled with an inhomogeneous medium where the interaction of diffusion, gravity, and acoustic radiation shapes a smooth gradient profile for the acoustic impedance of the media (*Z*_*m*_) orthogonal to the flow. In the stop-flow format of the IAF, a proper inhomogeneous medium is obtained by laminating two media of acoustic impedance higher and lower than that of the cells. After stopping the flow, in response to the sound field activation, the acoustic body force quickly relocates and stabilizes the medium of higher acoustic impedance against gravity. The solute iodixanol molecules in the center of the resonant acoustic field diffuse sideways, and thereby the acoustic impedance gradient slowly flattens until the two media are thoroughly mixed. Meanwhile, the acoustic radiation force (*F*_rad_) pushes the cells to a place where the acoustic contrast (Φ) and *F*_rad_ become zero, and thus the acoustically induced sideways velocity drops to zero. Figures 1(b) and 1(c) show schematically a cell approaching its IAP from its initial location in a low-impedance medium.

To adjust the acoustic impedance of the media, Optiprep™ (Stemcell Technologies, Norway) containing 60% w/v iodixanol was used as an agent to prepare the solutions of low and high acoustic impedance by adding a required amount of the Optiprep™ to PBS. Iodixanol has a high acoustic impedance to viscosity ratio and allows a substantial change in acoustic impedance with only modest changes in viscosity. The acoustic impedance of iodixanol solutions increases monotonically with the concentration when using isotonic serum-supplemented saline solutions such as PBS. PS particles and stained cells were suspended in the low acoustic impedance media (PBS, iodixanol 10%, and iodixanol 20%) and were introduced to the channel via the side inlets. The central inlet always contains a medium of high acoustic impedance (iodixanol 35%) mixed with 3 μg ml^–1^ of the fluorescent tracer molecule (Dextran, Cascade Blue™, 3000 MW, ThermoFisher). No tracer molecules were added to the low acoustic impedance medium.

For the experiments, the two liquids were injected into the channel with the same flow rate. The syringe pump’s unit associated with the central inlet was set to infuse at the flow rate of 100 μl/min, and the other unit at the outlet was programmed to withdraw and collect the entire flow with the flow rate of 200 μl/min. At time *t =* 0 s, the flow was stopped, the ultrasound was activated, and the image acquisition was started, repeatedly acquiring images of the particle or cell motion and the fluorescent dextran intensity profile. For each measurement set, the images were recorded for 100 s.

The entire procedure for image acquisition is similar to what we did in our previous study [34] and can be summarized in the following steps:

1. Inject cells suspended in a homogenous medium, stop the flow, and wait for cells to float toward the channel ceiling or sink to the channel floor.

2. Acquire a *z*-stack of calibration images of the cells at rest at either the ceiling or the floor.

3. Inject cells suspended in an inhomogeneous medium, stop the flow, start multichannel image acquisition, and activate the sound at a fixed *z*-position.

4. Acquire cell and gradient images at 10 fps for 100 s.

5. Acquire background and reference images where the channel is filled with the central stream at 10 fps for 100 s.

### D Tracking cells and particles

To record three-dimensional trajectories of PS particles and stained cells, we used defocusing-based particle tracking [43–45], which we have previously validated for use with cells [34]. Defocused microscope images of particles and cells at different depth positions are stored in a reference library and map the *z*-component of the trajectory for each particle and cell by cross-correlation with the library images. See further details in Ref. [34].

### E Mapping the acoustic impedance of the fluid

To map *Z*_*m*_ in space and time, we added a fluorescent dextran to the high-impedance medium. Under the assumption that iodixanol and dextran molecules diffuse at the same rate (*D*_iodixanol_ ≈ 2.5 × 10^−10^ m^2^ s^−1^ and *D*_dextran_ ≈2.2×10^−10^ m^2^ s^−1^), the two molecules are expected to maintain their relative proportion everywhere in the system [40,46]. Furthermore, in Ref. [40], it was established that dye intensity changes linearly with the iodixanol concentration when diluting the mixture with water, such that the iodixanol concentration (*C*_ix_) can be retrieved through the equation

(1) $$C_{\text{ix}}\left(I_{\text{norm}}\right)=C_{\text{ix}}^{\min}+I_{\text{norm}}\left(C_{\text{ix}}^{\max}-C_{\text{ix}}^{\min}\right).$$

**FIG. 2:**
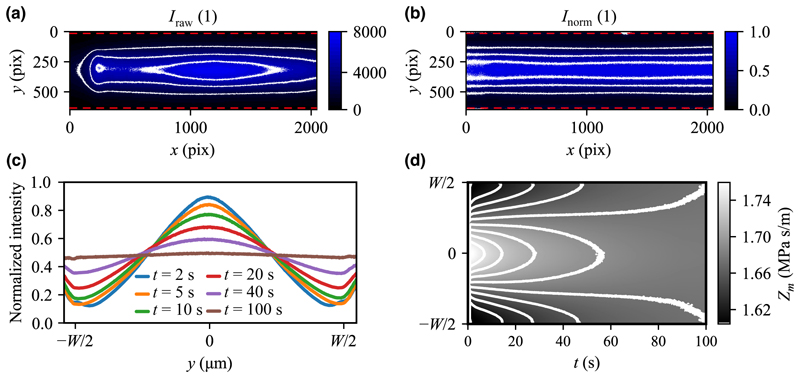
Mapping the acoustic impedance over time for *Z*_*m*_ = 1.58 to 1.77 MPa s/m. (a) The raw intensity image at *t* = 2 s and (b) the corresponding normalized image, with linearly spaced contour lines indicated in white. The red dashed lines in (a),(b) define the channel walls. (c) The time-dependent evolution of the concentration field with the acoustics present. Profiles show the median values taken along the channel length direction (*x*) from the normalized images. (d) Map of the development of the measured *Z*_*m*_(*y, t*) with gray levels indicating the acoustic impedance and linearly spaced contours.

Here, $C_{\text{ix}}^{\min}$ and $C_{\text{ix}}^{\max}$ are the iodixanol concentrations of the low (side inlet) and high (central inlet) impedance media, respectively. *I*_norm_= (*I*_raw_ − *I*_bg_)/(*I*_max_ − *I*_bg_ I _raw_) after having been background-subtracted and normalized using *I*_max_ and I_bg_ [Figs. 2(a) and 2(b)]. *I*_max_ is the image of a channel completely filled with the high-impedance medium, and *I*_bg_ is the image of the low-impedance zero-tracer medium. The normalization scheme is essential to our measurement procedure since it corrects the nonuniform microscope illumination and existing shadow effects in the imaging field [Fig. 2(a), left side]. To cancel the effect of photobleaching, *I*_bg_ and *I*_max_ were recorded under identical conditions as *I*_raw_, and the corresponding images in the sequence were used for the normalization (Appendix A).

Figure 2(c) shows the time-dependent evolution of the intensity field from a steep distribution at early times to a progressively more flat distribution at later times. The normalized intensity profiles here represent the median values taken along the channel length.

From the recorded intensity images *I*_norm_(*x, y, t*), the density (*ρ*_*m*_), compressibility (*κ*_*m*_), acoustic impedance (*Z*_*m*_), and viscosity (*η*) were computed. The scalar field for a property (*X*_*i*_) was derived based on a fitted polynomial (*f*_*i*_) to *a priori* measured data for *X* as a function of *C*_ix_ [%], such that *X*_*i*_ (*f*_*i*_[*C*_ix_(*I*_norm_[*x, y, t*])]), following the procedure and polynomials as in [40].

(2a) $$\rho_{m}\left(f_{\rho }\left[C_{\text{ix}}\right]\right)=1005-5.245C_{\text{ix}},$$

(2b) $$\kappa_{m}\left(f_{\kappa }\left[C_{\text{ix}}\right]\right)=4.384\times 10^{-10}-1.853\times 10^{-12}C_{\text{ix}}+3.116\times 10^{-15}C_{\text{ix}}{\text{‌}}^{2},$$

(2c) $$\begin{array}{c} \eta \left(f_{\eta }\left[C_{\text{ix}}\right]\right)=9.540\times 10^{-4}+1.952\times 10^{-5}C_{\text{ix}}+2.419\\ \times 10^{-7}C_{\text{ix}}{\text{‌}}^{2}+2.173\times 10^{-8}C_{\text{ix}}{\text{‌}}^{3}, \end{array}$$

(2d) $$Z_{m}=\sqrt{\rho_{m}/\kappa_{m}}$$

Figure 2(d) shows the development of the acoustic impedance profile over time [*Z*_*m*_(*y, t*)] based on the intensity profiles shown in Fig. 2(c) and Eqs. (1) and (2). This type of reduced mapping (i.e., collapsed along *x*) was used as input when generating synthetic particle and cell trajectories to enable faster interpolation. Appendix B describes the analysis of the artifacts in the gradient images.

### F Generating synthetic particle and cell trajectories

A numerical procedure was set up to understand better and highlight the main factors governing cell migration in acoustic impedance gradient media. Synthetic cell and particle trajectories were generated by modifying a previously described method where spherical particles are propelled by gravity and acoustic effects in a halfwavelength one-dimensional resonance [47]. We assumed that (i) cells are spherical and have homogeneous density and compressibility, (ii) gravity acts on the cells in the negative *z*-direction, (iii) the acoustic field and the acoustic impedance gradient are invariant in *x* and *z*, (iv) the acoustic radiation force on cells can be modeled by the classical Gor’kov theory [48], with a thermoviscous correction [49], (v) the acoustic streaming generated at the channel floor and ceiling is completely suppressed by a stabilizing acoustic body force due to the acoustic impedance gradient [37,38], (vi) the acoustic energy density (*E*_ac_) is constant, and (vii) the acoustic contrast factor (Φ) of a cell, in space and time, depend on the properties of the media at the location of the cell. We ignored wall effects on the Stokes drag force acting on the cells.

To realistically represent the slowly diffusing acoustic gradient medium in the model, we utilized a time series of normalized fluorescence intensity gradient images retrieved from the corresponding experimental datasets [Fig. 2(d)]. Intensity profiles *I*_norm_(*y, t*) were stored in a matrix of *y* and *t* and then were extracted for a requested location and time by interpolation using the MATLAB routine griddedInterpolant.

To generate a trajectory, we first specify *E*_ac_, and the cell’s starting position (*y*_*c*_, *z*_*c*_), radius (*a*), density (*ρ*_*c*_), and compressibility (*κ*_*c*_). Then we compute the cell location *y*_*c*_(*t*) and *z*_*c*_(*t*) as a function of time based on an explicit Runge-Kutta formula which is implemented in the MATLAB standard routine ode45 [50]. The main modification compared to our previous work [34] is that in the function to compute the velocity component (*u*_*y*_) of a cell for a given position and time, we now derive the time- and space-dependent Φ (*ρ*_*c*_, *κ*_*c*_, *ρ*_*m*_[*y*_*c*_(*t*)], *κ*_*m*_[*y*_*c*_(*t*)], *η*[*y*_*c*_(*t*)]) using

(3a) $$f_{1}=1-\kappa_{c}/\kappa_{m},$$

(3b) $$f_{2}=\frac{(2+3\delta /a)\left(\rho_{c}/\rho_{m}-1\right)}{2\rho_{c}/\rho_{m}+1+9\delta /2a},$$

(3c) $$\text{Φ}=f_{1}/3+f_{2}/2,\;$$

(3d) $$F_{\text{rad}}=-4E_{\text{ac}}k_{y}\pi a^{3}\text{Φ}\sin\left(2k_{y}y_{c}\right),$$

(3e) $$u_{y}=F_{\text{rad}}/6\pi \eta a,$$

(3f) $$u_{z}=2\left(\rho_{c}-\rho_{m}\right)ga^{2}/9\eta .$$

For Ф, we compute the viscous boundary layer thickness $\delta =\sqrt{\eta /\rho_{m}\pi f}$, and *k*_*y*_ is the wave number.

## III Results

### A Determination of the acoustic energy density

**FIG. 3:**
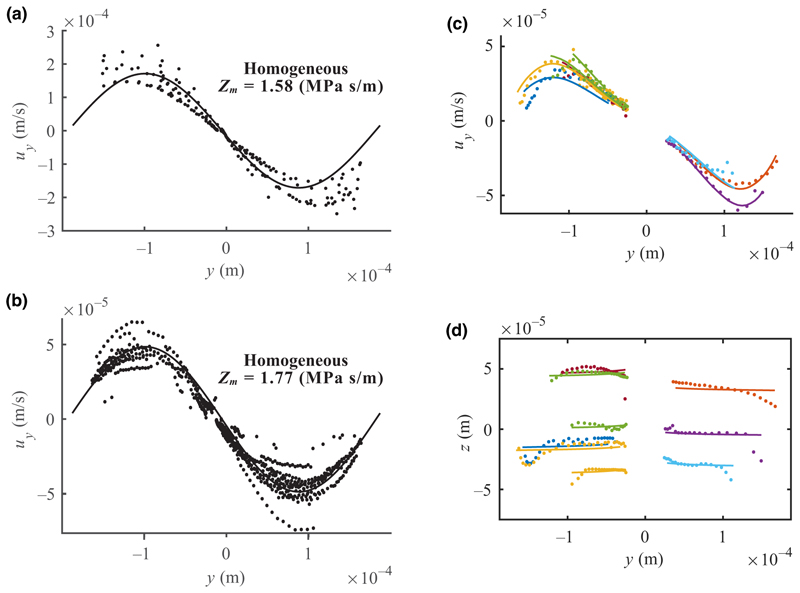
Velocity versus *y* for 4.9-μm-diameter PS particles in the homogenous medium of (a) *Z*_*m*_ = 1.58 MPa s/m, (b) *Z*_*m*_ = 1.77 MPa s/m, and (c) for a gradient of *Z*_*m*_ = 1.58 to 1.77 MPa s/m. (d) Positions of PS particles from *t* = 2 to 10 s in the *y*–*z* plane. In (a),(b) solid lines indicate sine curve fits. In (c),(d) every second experimental observation is indicated by a dot, and solid lines indicate simulated trajectories based on the search algorithm. Multiple particles can have the same color.

To estimate *E*_ac_ from the experiments as input to the numerical model of cell trajectories, we tracked 4.9-μm PS particles and employed two different approaches. In the first approach, introduced and explained in [34], *E*_ac_ was evaluated in homogeneous media by fitting the velocity data of particles (*u*_*y*_) near the mid-height in the channel to an analytical model that considers the acoustic radiation force and the acoustic streaming. Figures 3(a) and 3(b) show that *u*_*y*_ decreases dramatically when *Z*_*m*_ increases from 1.58 to 1.77 MPa s/m (homogeneous) due to a higher viscosity and a slightly lower acoustic contrast, but the measured *E*_ac_ is seemingly unchanged when changing the medium, and the measurement is consistent within 10% for particles of different sizes (Supplemental Material, Fig. S1 [51]). Further, our analysis confirms that *E*_ac_ increases, as expected, linearly with the square of the piezo actuator voltage peak-to-peak amplitude $\left(U_{\text{pp}}^{2}\right)$ up to *E*_ac_ ≈ 45 J m^–3^. PS particles have relatively similar acoustic contrast in both homogeneous media (*Z*_*m*_ = 1.58 and 1.77 MPa s/m) due to their much smaller compressibility; therefore, their migration is relatively more affected by the increasing viscosity.

To understand if *E*_ac_ measured in homogeneous media can predict *E*_ac_ for inhomogeneous acoustic impedance gradients, we analyzed trajectories of PS particles in an acoustic impedance gradient spanning from *Z*_*m*_ = 1.58 to 1.77 MPa s/m for identical actuation settings as in the homogeneous case. Figure 3(c) shows that *u*_*y*_ for the inhomogeneous case falls slightly below the homogeneous systems in Figs. 3(a) and 3(b), while it was expected to fall in between the two, indicating a lower *E*_ac_. To investigate this, we analyzed the trajectories in the *y*–*z* plane to find the corresponding best-matching synthetic trajectory when employing the MATLAB function fminsearch using *E*_ac_ and the particle’s starting position as free-fitting parameters (Appendix C). The fact that the fitted trajectories match the experimental trajectories with an average position error of 3.87 μm [standard deviation (SD) 2.05 μm], which corresponds to approximately one bead diameter or one-hundredth of the channel width, makes us confident that the model describes the system reasonably well [Fig. 3(d)]. The average resulting *E*_ac_ was 23.4 J/m^3^ (SD 4.64 J/m^3^) which is considerably lower than what we found for the measurements in homogeneous media, and this value will be used in the following when modeling cell trajectories. A likely cause of the discrepancy is linked to an altered resonance due to the inhomogeneity [52]. Therefore, we performed a one-dimensional eigen-frequency analysis of homogeneous and inhomogeneous fluids in Comsol Multiphysics (Appendix D). The analysis shows a considerable shift of the resonance frequency *f*_0_ for the inhomogeneous system that we do not see for the corresponding homogenous fluids, and this can explain why identical drive parameters can lead to a large change in *E*_ac_.

### B Acoustic streaming suppression

**FIG. 4:**
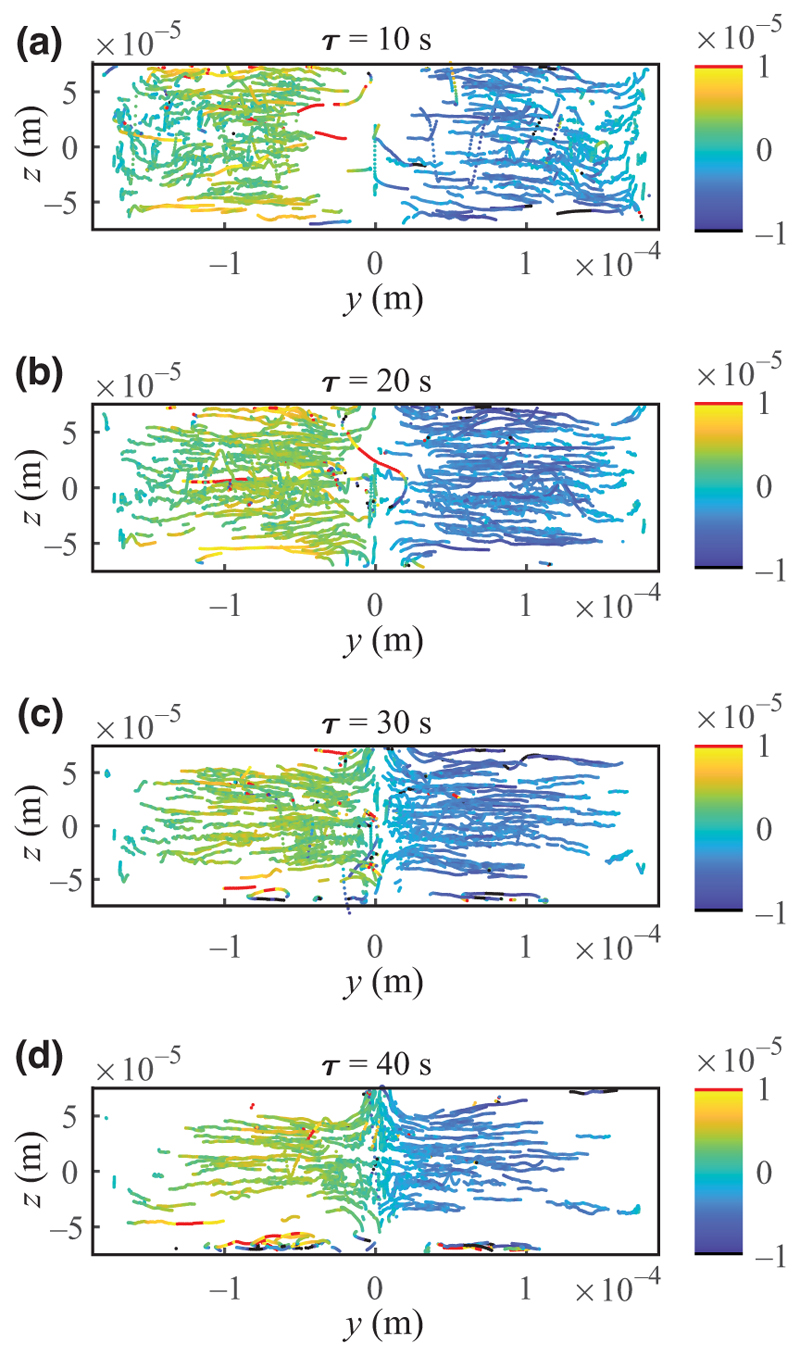
Experimental trajectories of 1.1-μm-diameter PS particles in the diffusing iodixanol gradient (*Z*_*m*_ = 1.58 to 1.77 MPa s/m) at mid-interval times (*τ*) of (a) 10 s, (b) 20 s, (c) 30 s, and (d) 40 s. Each interval corresponds to 10 s. The color scale indicates *u*_*y*_ (m/s), with black and red showing velocities outside the range.

Cells migrating in an acoustic field in a homogeneous fluid at near-zero acoustic contrast are highly affected by acoustic streaming [34]. However, previous works have shown that acoustic streaming can be suppressed in the presence of an acoustic impedance gradient [37,38], and therefore in the present paper we have omitted acoustic streaming in the numerical model. To test this assumption, we tracked 1.1-μm-diameter PS tracer particles to investigate the presence of acoustic streaming and its potential impact on the cell measurements in a diffusing iodixanol gradient from *Z*_*m*_ = 1.58 MPa s/m (sides) to 1.77 MPa s/m (center). Figure 4 shows that the acoustic streaming is substantially suppressed in the bulk of the channel at early times, and hence the trajectories of the tracer particles do not follow the four quadrupolar streaming rolls, which is the typical motion of particles smaller than the crossover size (approximately 2 μm) in homogenous media (Supplemental Material, Fig. S2 [51]). Instead, the particles in the bulk slowly become focused to the center of the channel, but after 30 s, particles that are close to the ceiling or floor start to form confined rolls due to the strong streaming near the horizontal boundaries and, in those regions, become depleted. As time evolves, the streaming rolls grow due to both diffusion and advection, and at late times when inhomogeneity vanishes, particles form typical streaming rolls similar to homogenous media. The analysis indicates that in the first 30 s or so, the streaming is efficiently suppressed in the bulk, and thus we assume our streaming-free model to be valid away from the floor and ceiling. Further, measuring the IAP of cells at times later than 30 s is problematic in this configuration since the cells may be dragged into the growing flow rolls where their position will not correctly match the properties of the surrounding medium. Consequently, cells registered outside the range abs(*z*_*c*_) *< W*/3 were omitted from further analysis. We also analyzed the streaming-free region for a diffusing iodixanol gradient from *Z*_*m*_ = 1.66 to 1.77 MPa s/m and observed a similar trend (Supplemental Material, Fig. S2 [51]).

### C Measuring the effective acoustic impedance of cells

**FIG. 5:**
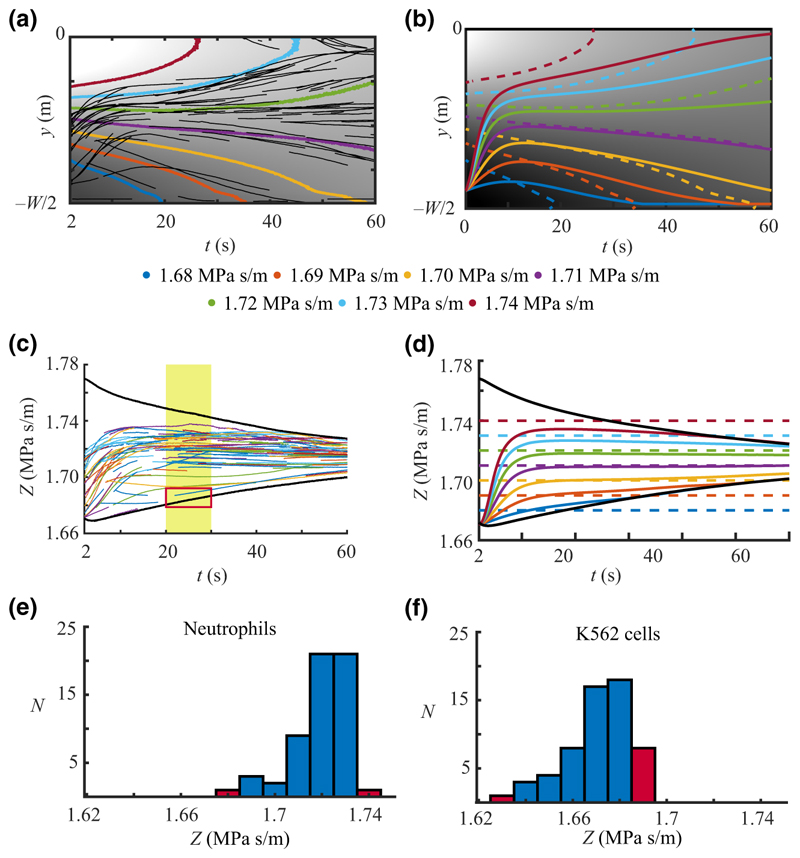
Experimental and simulated cell trajectories. (a) Neutrophil *y*-coordinate versus time in an acoustic impedance gradient (black curves). *Z*_*m*_ is indicated by grayscale values spanning from 1.64 MPa s/m (black) to 1.75 MPa s/m (white). Colored contours indicate constant *Z*_*m*_. (b) Plot of simulated trajectories *y*_*c*_(*t*) for cells of hypothetical *Z*_*c*_ (thick colored lines) in the same acoustic impedance gradient as in (a). *Z*_*m*_ is indicated by grayscale values spanning from 1.68 MPa s/m (black) to 1.74 MPa s/m (white). (c) Experimentally measured effective acoustic impedance of neutrophils versus time when assigning *Z*_*c*_ = *Z*_*m*_ (multiple cells can have the same color). Solid black lines indicate *Z*_*m*_ at the channel walls (lower line) and center (upper line). The yellow region shows the time span of the measurement, and the red box highlights a cell with properties outside the measurable range. (d) Simulated *Z*_*m*_ (solid lines) and true *Z*_*c*_ (dashed lines) for hypothetical cells. (e) Distribution of the measured effective acoustic impedance of neutrophils in the time interval from 20 to 30 s after the onset of sound in a gradient ranging from *Z*_*m*_ = 1.66 to 1.77 MPa s/m, and (f) the corresponding distribution for K562 cells measured in a gradient ranging from *Z*_*m*_ = 1.51 to 1.77 MPa s/m. In (e),(f), red bars indicate cells registered near the center or sides outside the measurable range.

To understand how cells reach their IAP, we first studied the migration of neutrophils in acoustic impedance gradients and compared it to synthetic cell trajectories. Figure 5(a) shows the *y*-position of cell trajectories against time for neutrophils for one half of the channel, overlaid on top of the measured acoustic impedance field of the medium *Z*_*m*_(*y, t*) indicated by grayscale and contours. Initially, cells move across the contours, but after 20 s, although still moving along *y*, their trajectories are aligned with the contours, indicating that they have reached their IAP. After 30 s, their deviations in *y* seemingly increase, which is confirmed by the simulated trajectories in Fig. 5(b). The reason is that near their IAP, cells experience weak acoustic radiation forces, and due to the molecular diffusion, the location of the IAP is accelerating along *y*. The optimal location *y*_*c*_ for a cell to stay in its IAP is if *Z*_*c*_ is close to the mean *Z*_*m*_ in the channel because that contour level is time-invariant and the location is near |y_c_| = W/4, which is where the factor sin(2*k*_*y*_ *y*_*c*_) in Eq. 3(d) has its maximum.

When assigning *Z*_*c*_ = *Z*_*m*_ and plotting against time, each trajectory converges to a constant value after about 20 s [Fig. 5(c)]. The impedance range gradually decreases with time due to diffusion, resulting in cells eventually reaching the center or sides of the channel. The simulated trajectories show a similar pattern in Fig. 5(d) and highlight that the approach of assigning *Z*_*c*_ = *Z*_*m*_ is associated with a systematic error which was previously investigated in [40]. In Appendix E, we simulate the error when measuring neutrophils of different *Z*_*c*_ for a range of *E*_ac_. Figure 5(e) shows the distribution of measured *Z*_*c*_ for neutrophils, evaluated in a timeframe from 20 to 30 s for a gradient *Z*_*m*_ = 1.66 to 1.77 MPa s/m. Cells registered near the center or sides are considered outside the measurement range and therefore marked with red bars.

We next studied the migration of K562 cells in an acoustic impedance gradient. Figure 5(f) shows the distribution of K562 cells’ effective acoustic impedance measured in a gradient of *Z*_*m*_ = 1.51 to 1.77 MPa s/m, indicating that they have lower and only slightly overlapping IAP distributions and thus are, to a high degree, separable in acoustic impedance gradients.

To illustrate the importance of using an appropriate acoustic impedance gradient, we also analyzed neutrophils’ migration in a gradient of *Z*_*m*_ = 1.58 to 1.77 MPa s/m. This concentration range causes *Z*_*m*_ to drop below *Z*_*c*_ before the cells reach their IAPs, and thus the high-impedance neutrophils fall outside the measurable range (Supplemental Material, Fig. S3 [51]).

The first 2 s after the onset of sound have been omitted from the experimental data due to a gravity effect. Before the image acquisition and sound actuation start, the high acoustic impedance medium in the center occupies the lower half of the channel due to its higher density. Upon sound actuation, the fluid quickly relocates to a stable configuration with high-impedance fluid at the center [35,36]. The timescale of this relocation is less than 1 s, whereas the cell migration towards the IAP is about 10 s, and thus, in the first moment after starting the sound, the cells follow their initial low-impedance fluid before starting to move toward their IAPs.

### D Cell separation in a continuous-flow gradient

**FIG. 6:**
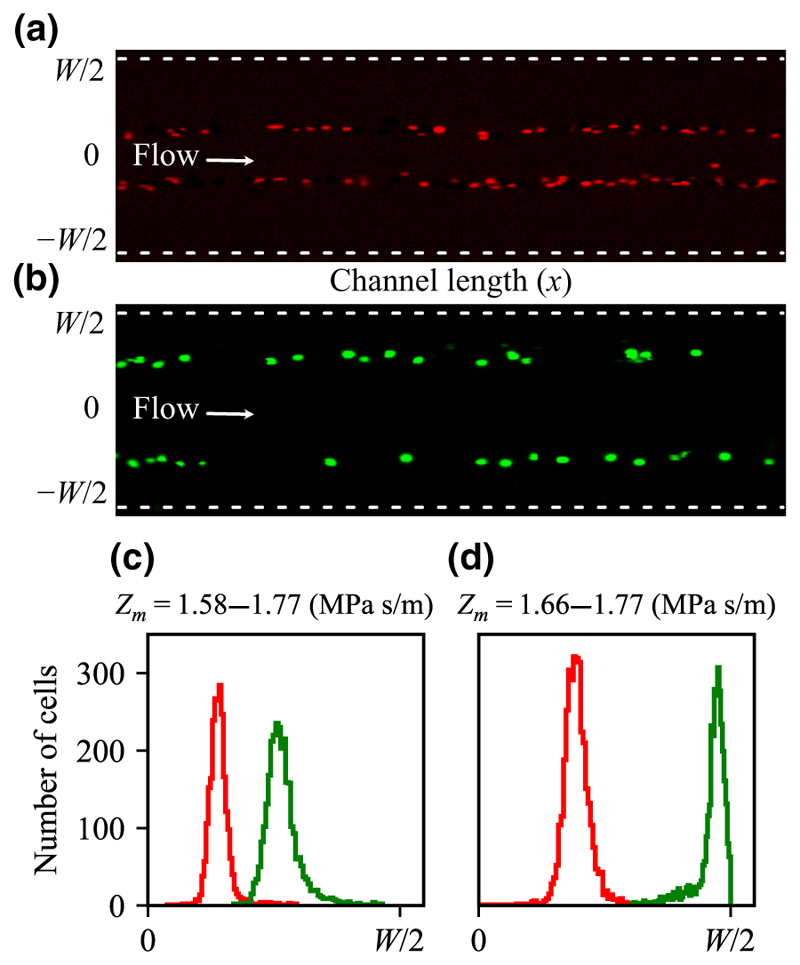
Neutrophils and K562 cells separated in an acoustic impedance gradient. (a) Neutrophils (red) near the end of the separation channel form bands at their IAP in a gradient formed by *Z*_*m*_ = 1.58 to 1.77 MPa s/m and (b) K562 cells (green) in the same gradient. (c),(d) Histograms of cell locations of neutrophils (red) and K562 cells (green) for gradients of (c) *Z*_*m*_ = 1.58 to 1.77 MPa s/m and (d) *Z*_*m*_ = 1.66 to 1.77 MPa s/m, respectively.

The results from the stop-flow measurements show that neutrophils and K562 cells have different effective acoustic impedances and that this difference should be sufficient to separate them from each other with minimal overlap [Figs. 5(e) and 5(f)]. To investigate if this separation can be implemented for continuous flow separation, we suspended a mixture of the two cell types in the side stream in a medium of either *Z*_*m*_ = 1.58 MPa s/m or *Z*_*m*_ = 1.66 MPa s/m. The central stream had *Z*_*m*_ = 1.77 MPa s/m and the total flow rate was 15 μl/min. Figures 6(a) and 6(b) show that neutrophils (red) and K562 cells (green), imaged in two different fluorescent channels, end up at different *y*-positions at the end of the channel. The distributions of the locations show that for a gradient *Z*_*m*_ = 1.58 to 1.77 MPa s/m, there is a slight overlap [Fig. 6(c)] which disappears for *Z*_*m*_ = 1.66 to 1.77 MPa s/m with the potentially undesired effect that K562 cells are being pushed into contact with the channel walls [Fig. 6(d)].

## IV Discussion

In this study, we established that there is a minimal overlap in the *Z*_*c*_ distributions of neutrophils and K562 cells by measuring their IAPs in acoustic impedance gradients in stop-flow. We then showed that neutrophils and K562 cells can be separated to a very high degree by their acoustic impedance in a flowthrough setting. By comparing to our previous study of these cell types in a range of homogeneous media [34], we can see that measurements in an acoustic impedance gradient have clear benefits. In that study, we systematically studied these cells in homogeneous media for a range of different *Z*_*m*_; we concluded that the presence of acoustic streaming for cells near zero acoustic contrast limits the ability to separate cells even if they have nonoverlapping distributions in *Z*_*c*_. For no medium was it possible to achieve positive contrast for a majority of one cell type while achieving negative contrast for the majority of the other. Here, the presence of the acoustic impedance gradient efficiently suppresses mixing by acoustic streaming, enabling both measurement and deterministic separation of the two cell types. Our experimental and numerical results indicate that IAF enables the determination of the IAP of cells if a set of conditions are met, and these are discussed below.

When configuring the acoustic impedance gradient, a tradeoff exists between having a large measurement range and achieving good precision in determining *Z*_*c*_. However, our analysis of the resonance shift in systems of layered fluids (Appendix D) indicates that using too large a range of acoustic impedance can complicate the measurements since the system may drift in or out of resonance during the measurement, leading in our case to lower *E*_ac_ than for the homogenous case. The shift agrees well with Ref. [52], where they predicted a 64 kHz shift in the resonance frequency in the case of strongly inhomogeneous fluids (10% acoustic impedance difference). It is worth mentioning, though, that such a resonant inhomogeneous system may just as well drift from high toward lower *E*_ac_ values depending on how the operating frequency is chosen. Moreover, since the gradient continuously evolves over time until reaching a homogenous state, it is likely that the *E*_ac_, measured within the first 10 s, slowly increased over the course of the measurement.

While acoustic streaming is suppressed in most parts of the acoustic chamber, it limits the IAP determination near the floor and ceiling of the IAF chamber. As shown in Fig. 4, acoustic streaming generates flat vortices near the channel floor and ceiling, which can drag cells out of position relative to the gradient image, which is recorded at the channel mid-height. The 30 s window for recording IAP is in line with previous investigations theoretically and experimentally [37,38].

Optical effects can lead to inaccurate determination of the IAP. One such effect is related to the presence of acoustic streaming near the channel floor and ceiling. The rotating fluid transports fluorescent molecules across the channel and may contribute to some extent to the gradient images, albeit not being in the optical focus. However, the objective has an optical slice thickness considerably smaller than the channel height [53], making this effect minimal. As shown in Appendix B [Fig. 8(c)], the insertion of the cylindrical lens in the optical system does not distort the image of the gradient in fluorescence intensity. The reason for this is that the lens was positioned such that its power direction coincides with the channel length direction (*x*). This assumes that the gradient is invariant, or at least slowly varying, along *x*, which is indeed the case in this study.

The cylindrical lens was primarily used to facilitate particle and cell tracking. The *z*-level reference library for the particle and cell-defocused images acquired in step 2 (Sec. II C) in homogenous media corresponds to both the side and center streams, which have different refractive indices (*n*_*s*_ = 1.34 and *n*_*c*_ = 1.37). However, the final image acquisition occurs for the inhomogeneous system where the refractive index changes with *y* [54], for which we had no way of generating reference images. A calibration graph for neutrophils suspended in media of *Z*_*m*_ = 1.58 MPa s/m and *Z*_*m*_ = 1.77 MPa s/m is shown in Supplemental Material, Fig. S4 [51]. It can be seen that the measured versus real *z* overlaps almost completely such that we can obtain accuracy in determining *z* within approximately one cell diameter, as established in Ref. [34].

Differences in the diffusion constant between the fluorescent dextran tracer molecules and the iodixanol molecules that make up the acoustic impedance gradient can lead to an inaccurate reading of the IAP. This results in a spatial mismatch between the measured fluorescent tracer intensity gradient and the acoustic impedance gradient. To further complicate matters, the solute iodixanol molecules are not dilute, and therefore the diffusivity of both iodixanol and the fluorescent dextran tracer molecules will depend on the local iodixanol concentration. Another related and entwined effect is that the iodixanol quenches the fluorescence signal of the tracer in a concentration-dependent manner. Thus, if the tracer diffuses faster than the iodixanol, it will reach a region of low concentration and appear brighter, leading to a further overestimation of the IAP at that point, or vice versa if the tracer is slower than the iodixanol. This effect can be observed in Appendix B [Fig. 8(c)], where a medium of high iodixanol and tracer concentration is sandwiched between low-iodixanol, tracer-free, media at high flow velocity. At the interface of the two fluids, the intensity increases, but since diffusion slows dramatically when the gradient in concentration of a molecule flattens, we do not observe this effect in the stop-flow experiments.

Here we measured neutrophils to have IAP ranging from 1.72 to 1.73 MPa s/m [Fig. 5(e)]. This falls between what was previously measured by tracking cell migration in homogenous media, 1.72 MPa s/m [34], and in a continuous flow acoustic impedance gradient, 1.74 MPa s/m [40]. For K562 cells, we measured IAP ranging from 1.67 to 1.68 MPa s/m, which is slightly higher than our previous study in homogenous media [34], 1.65–1.67 MPa s/m, and in close agreement with a study by Olofsson *et al*. [31], 1.67–1.70 MPa s/m. The density of living cells is likely donor-dependent and can change due to preparation and culture conditions, and we could not, therefore, conclude whether the discrepancies are caused by technical inaccuracies or underlying cell properties. Even though the stopped-flow format is disadvantageous compared to the continuous flow method in terms of throughput, it provides detailed information on single cells’ trajectories in a gradient of acoustic impedance and can possibly be used to extract additional properties such as sedimentation in gravity. Looking ahead, evaluation and benchmarking of systems for separation of cells at near-zero acoustic contrast would be of great benefit if suitable calibration particles were available having appropriate compressibility, density, and size.

## V Conclusions

We studied the trajectories of cells in diffusing iodixanol gradients to measure their effective acoustic impedance. We set up a simple numerical model and used microparticles to estimate the acoustic energy density in the layered configuration of fluids. We showed that the model could predict the experimental cell trajectories and measure the IAP of cells with good qualitative agreement. The streaming-free environment enabled by the acoustic impedance gradients makes the current approach beneficial compared to the separation of cells in a homogeneous medium of tailored acoustic impedance.

## Supplementary Material

Supplementary information

Appendices
