# Supplementary material for: Acoustophoretic Characterization and Separation of Blood Cells in Acoustic Impedance Gradients
Source: Phys Rev Appl. Author manuscript; Available in PMC 2024 Feb 8. (PMC7615610; doi:10.1103/PhysRevApplied.20.024066)
Supplement: Supplementary information [file EMS193827-supplement-Supplementary_information.pdf]

Supplemental material for paper:

Acoustophoretic Characterization and Separation of Blood Cells in Acoustic Impedance Gradients

Mahdi Rezayati Charan and Per Augustsson*

Department of Biomedical Engineering, Lund University, Ole Römers väg 3, 22363 Lund, Sweden

* per.augustsson@bme.lth.se

S1. Acoustic energy density in homogeneous media

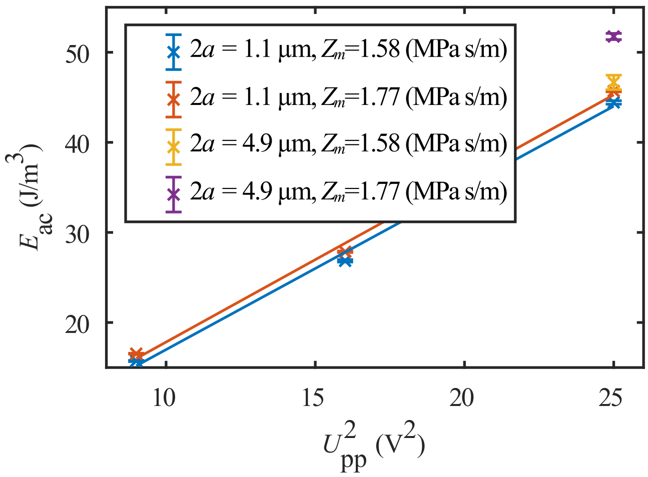

FIG. S1. Acoustic energy density estimations in homogeneous media for increasing piezo actuator voltage amplitude (U_{pp}) for PS beads of diameter 1.1 µm and 4.9 µm and Z_m = 1.58 and 1.77 MPa s/m. Marker (×) indicates fitted E_{ac}, and bars indicate E_{ac}'s upper and lower confidence levels. Solid lines represent linear fits through fitted E_{ac} vs. U_{pp}^2.

S2. Three-dimensional trajectories of 1.1-μm-diameter PS particles

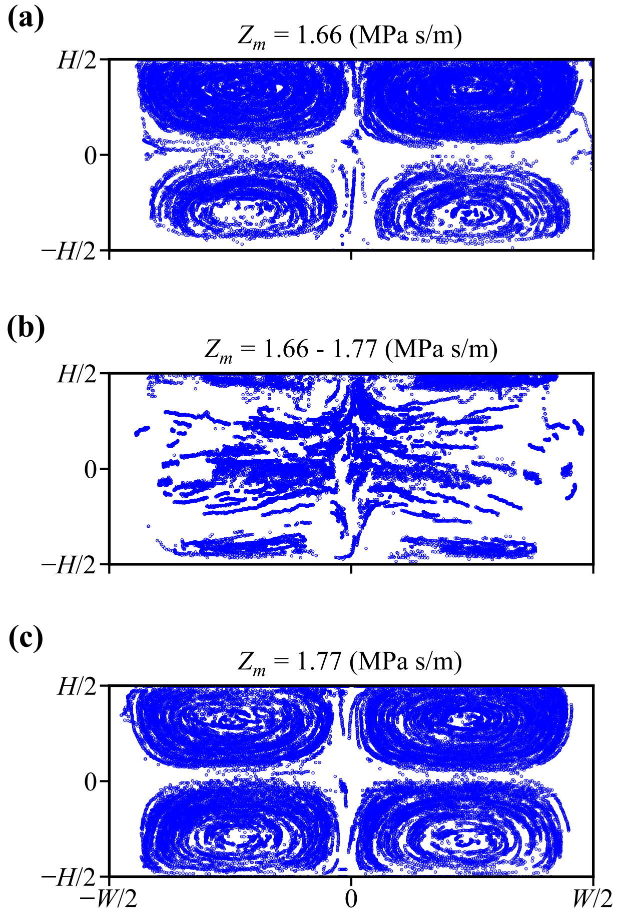

FIG. S2. Experimental trajectories of 1.1 μm in diameter PS particles at the mid-interval time (τ) 40 s in (a) homogenous medium of Z_m = 1.66 MPa s/m, (b) a gradient from Z_m = 1.66 to 1.77 MPa s/m, and (c) homogenous medium of Z_m = 1.77 MPa s/m.

S3. Neutrophils in a gradient of Z_m = 1.58 to 1.77 MPa s/m

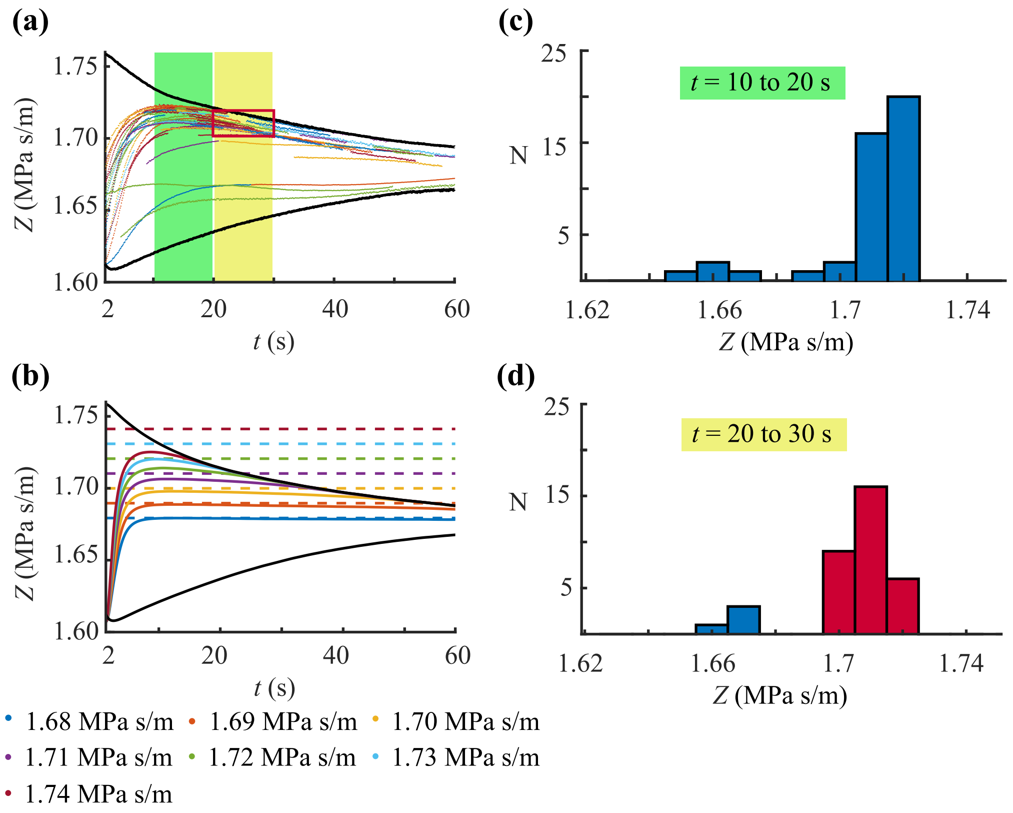

FIG. S3. (a) Experimentally measured the effective acoustic impedance of neutrophils vs. time when assigning $Z_c = Z_m$. Multiple neutrophils can have the same color. Solid black lines indicate Z_m at the channel walls (lower line) and center (upper line). The green and yellow regions show the time span of the measurement, and the red box highlights neutrophils with properties outside the measurable range. (b) Simulated Z_m (solid lines) and true Z_c (dashed lines) for hypothetical cells. The solid black lines indicate the same Z_m as in (a). Distribution of the measured effective acoustic impedance of neutrophils in the time interval (c) 10 to 20 s and (d) 20 to 30 s after sound onset. Red bars indicate cells registered near the center or sides, outside the measurable range.

S4. Measuring neutrophils' z-coordinate

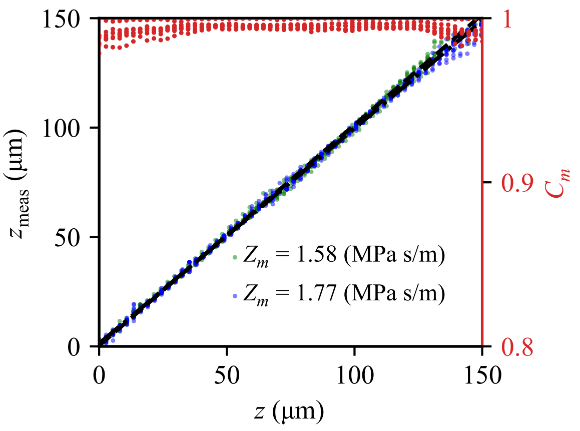

FIG. S4. A plot of z_{meas} and correlation coefficient C_m (red dots) versus the microscope-stage z-coordinate for neutrophils suspended in the homogeneous medium of Z_m = 1.58 MPa s/m (green dots) and Z_m = 1.77 MPa s/m (blue dots). The fitted lines (black) almost overlap. The data for neutrophils in Z_m = 1.77 MPa s/m comes from our previous study [34].

References

[34] M. Rezayati Charan, F. Berg, and P. Augustsson, Acoustofluidic three-dimensional motion of suspended cells at near-zero acoustic contrast in homogeneous media, Physical Review Applied **19**, 014046 (2023).
