# Supplementary material for: Acoustophoretic Characterization and Separation of Blood Cells in Acoustic Impedance Gradients
Source: Phys Rev Appl. Author manuscript; Available in PMC 2024 Feb 8. (PMC7615610; doi:10.1103/PhysRevApplied.20.024066)
Supplement: Appendices [file EMS193827-supplement-Appendices.pdf]

donor-dependent and can change due to preparation and culture conditions, and we could not, therefore, conclude whether the discrepancies are caused by technical inaccuracies or underlying cell properties. Even though the stopped-flow format is disadvantageous compared to the continuous flow method in terms of throughput, it provides detailed information on single cells' trajectories in a gradient of acoustic impedance and can possibly be used to extract additional properties such as sedimentation in gravity. Looking ahead, evaluation and benchmarking of systems for separation of cells at near-zero acoustic contrast would be of great benefit if suitable calibration particles were available having appropriate compressibility, density, and size.

V. CONCLUSIONS

We studied the trajectories of cells in diffusing iodixanol gradients to measure their effective acoustic impedance. We set up a simple numerical model and used microparticles to estimate the acoustic energy density in the layered configuration of fluids. We showed that the model could predict the experimental cell trajectories and measure the IAP of cells with good qualitative agreement. The streaming-free environment enabled by the acoustic impedance gradients makes the current approach beneficial compared to the separation of cells in a homogeneous medium of tailored acoustic impedance.

ACKNOWLEDGMENTS

We are grateful to Dr. Rune Barnkob (Technical University of Munich, Germany) and Dr. Massimiliano Rossi (University of Bologna, Italy) for providing the software for particle tracking. The project was funded by the Swedish Foundation for Strategic Research (Grants No. ICA16-0002 and No. FFL18-0122) and European Research Council (ERC) under the European Union's Horizon 2020 Research and Innovation Programme (Grant Agreement No. 852590).

APPENDIX A: COMPENSATING GRADIENT IMAGES FOR PHOTOBLEACHING

Since the flow is stopped and the channel is illuminated for a relatively long period of time, it was necessary to compensate for the gradient images due to photobleaching. To illustrate the effect, a sequence of gradient images was analyzed by taking the average along one pixel row in the length direction at the center of the channel. First, this was done for a completely filled channel (I_{max}), which is expected to be invariant over time if there is no photobleaching. Figure 7(a) (blue line) shows a clear drop in the measured intensity over time when we used I_{max} at time 0 to normalize all consecutive images.

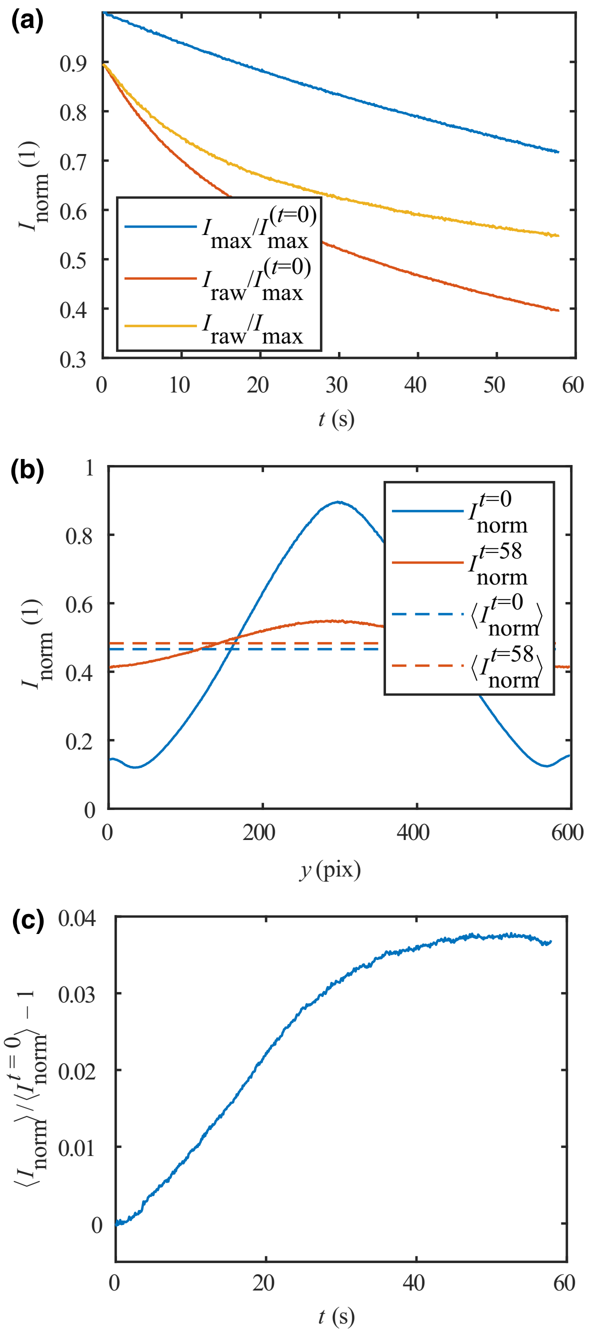

FIG. 7. (a) Median intensity as measured along the center pixel row in the channel for the I_{max} images normalized using I_{max} at $t = 0$ s (blue), for I_{raw} normalized using I_{max} at $t = 0$ s (red), and for I_{raw} normalized using I_{max} for the corresponding timepoint (yellow). (b) Intensity profiles at times 0 s and $t = 58$ s (solid lines) and their corresponding averages (dashed lines). (c) Relative error in the averaged normalized fluorescent intensity in one field of view over time as compared to $t = 0$ s.

When analyzing an impedance gradient image sequence, diffusion and bleaching are expected to contribute to the decaying intensity when normalizing I_{raw} using I_{max} at time 0 (red line). When instead the sequence of I_{max} images

was used to normalize $I_{\rm raw}$ for the corresponding time, the effect of photobleaching seemingly disappeared. If the normalization works, the prediction would be that over the course of an experiment, the mean intensity of a complete profile spanning the whole channel would be expected to be unchanged due to the conservation of molecules in the field of view. Figure 7(b) shows the normalized profiles at $t = 0$ and 58 s and their mean values. Only a slight shift is observed, and Fig. 7(c) shows the error development for the normalized mean intensity over time, indicating an overall overestimation of the intensity values below 4%.

APPENDIX B: ANALYSIS OF ARTIFACTS IN THE GRADIENT IMAGES

When analyzing cells' acoustic impedances in the experiments, the entire dataset for $I_{\rm norm}(x, y, t)$ was used since the acoustic field in the field of view varies slightly across the channel length. To smooth the intensity profile across the channel length, a Gaussian filter was implemented to reduce the noise level. Minor errors associated with the smoothing are considered when we read the acoustic impedance in the corresponding location of cells. Due to a slight overlap between the emission spectrum of the PS particles, stained cells, and fluorescent dextran, the particles tend to bleed into the fluorescent dextran intensity images $I_{\rm norm}(x, y, t)$. To remove the particle images from the gradient images, a mask is produced by thresholding the corresponding particle image and this mask is then used to mask out the particle from the gradient image. Finally, the MATLAB routine `fillmissing` was used to repair the gradient images [Figs. 8(a) and 8(b)].

We used a cylindrical lens to record defocused images of particles and cells for particle tracking. The lens was oriented to focus light in the x-direction, that is, along the channel, to minimize the distortion to the imaging of the gradient. We imaged the channel for a continuous-flow regime where two liquids ($Z_m = 1.58$ MPa s/m in the side inlet and $Z_m = 1.77$ MPa s/m in the center inlet) were laminated side by side. The total flow rate was 200 µl/min, and the flow ratio was fixed at around 1. The objective was placed at the mid-height, and the median value across the channel length was used to plot the intensity profile. Figure 8(c) shows that the acquired gradient profiles with and without the cylindrical lens overlap almost perfectly.

To verify the assumption that the gradient can be treated as invariant in the z-direction, we acquired gradient images at different heights for the gradient ranging from $Z_m = 1.58$ to 1.77 MPa s/m. The cylindrical lens was inserted, and the objective was placed at three different heights of lower height ($z = 37.5$ µm), mid-height ($z = 75$ µm), and upper height ($z = 112.5$ µm). Figures 8(d) and 8(e) show that the intensity profile is maintained with only slight discrepancies for low and high $E_{\rm ac}$, respectively.

Finally, since photobleaching is present during the experiments, we investigated images where the channel is filled with only the central medium, with and without

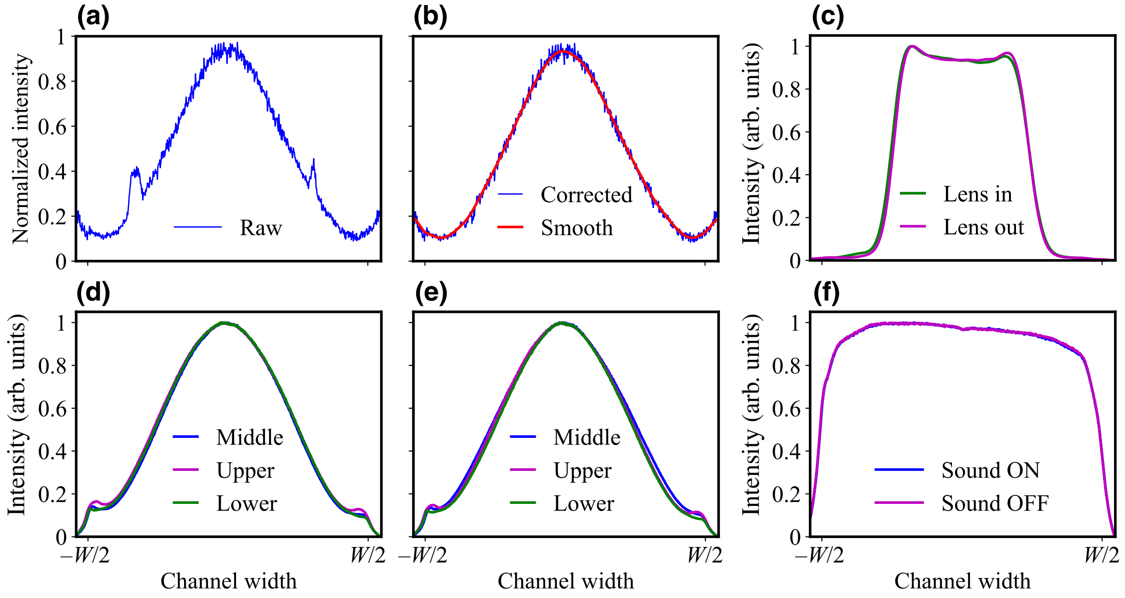

FIG. 8. (a) Normalized intensity profile at x across the channel length (L) where part of the cells is bleeding through the corresponding gradient image. The profile belongs to the normalized gradient image recorded at $t = 2$ s. (b) Plot of the corrected and smoothed intensity profile. The Gaussian filter was applied to smooth the corrected profile. (c) Gradient profiles for two cases: the cylindrical lens is placed in and out of the light path. With the cylindrical lens inserted, the intensity profile at different heights is shown at (d) low and (e) high $E_{\rm ac}$ for the corresponding gradient image at $t = 2$ s. (f) Plot showing the median value across the channel length for the reference images at $t = 50$ s. The optical focus is placed at the channel mid-height.

TABLE I. Result of fitting E_{ac} in the inhomogeneous medium.

Trajectory	E_{ac} (J/m^3)	Position error (μm)
1	20.2	1.81
2	27.3	2.09
3	21.5	3.33
4	25.3	4.42
5	31.7	3.00
6	25.2	3.18
7	15.7	7.93
8	20.6	6.43
9	22.9	2.69
**Mean**	23.4	3.87
**SD**	4.64	2.05

activated ultrasound, to verify that the acoustic streaming in the homogenous medium does not affect the intensity profile [Fig. 8(f)].

APPENDIX C: ESTIMATING E_{ac} BY FITTING SYNTHETIC TRAJECTORIES TO EXPERIMENTAL TRAJECTORIES

To estimate E_{ac} for the inhomogeneous system, we used the function `fminsearchbnd`, which is based on the built-in MATLAB optimization function `fminsearch` [55]. As input, the cost function to minimize takes the experimental particle trajectory, the optimization parameters E_{ac}, y_0, and z_0, and maps of the viscosity, density, and compressibility in space and time. In each iteration, the cost function generates a synthetic trajectory based on the optimization parameters and computes the average of the Euclidean distances between observed and synthetic positions over time. Table I shows the fit of experimental trajectories in a diffusing iodixanol gradient from $Z_m = 1.58$ to 1.77 MPa s/m and for $U_{pp}^2 = 25 V^2$ when using E_{ac}, $y_{(t=2)}$, and $z_{(t=2)}$ as free-fitting parameters.

APPENDIX D: EIGENFREQUENCY ANALYSIS OF LAYERED FLUIDS

A Comsol eigenfrequency simulation (pressure acoustics, frequency domain) was conducted to find the idealized one-dimensional fundamental resonance frequencies for homogeneous and smoothly inhomogeneous media. $C_{ix}(y)$ was set to vary between C_{ix}^{side} and C_{ix}^{center} as a Gaussian pulse (built-in) of standard deviation $W/6$, and the corresponding ρ_m, c_m, and Z_m were then derived. Figure 9 shows the synthetic acoustic impedance profiles that were assumed in the simulation. Table II shows that (i) the resonance frequency of layered fluids does not fall inside the range of the resonance frequencies of the two corresponding homogeneous fluids, and (ii) the frequency shift increases (with a negative sign) with increasing differences in C_{ix} between the side and central fluid.

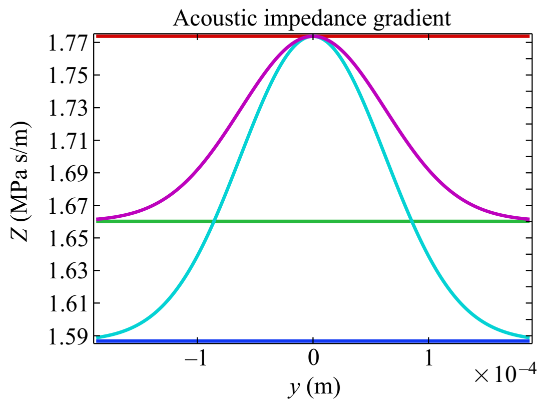

FIG. 9. Synthetic acoustic impedance (Z) profiles across the channel width used in the eigenfrequency analysis for homogeneous media of $C_{ix} = 10\%$ (blue), 20% (green), and 35% (red), and for smoothly inhomogeneous media of $C_{ix} = 10\%$ to 35% (cyan) and 20% to 35% (magenta).

APPENDIX E: CELL IAP ERROR ESTIMATION

Figure 10(a) shows the relative error $(Z_{meas} - Z_c)/Z_c$ when assigning $Z_c = Z_m$ for the synthetic trajectories shown in Fig. 5(d). The error has two parts which are highlighted in Figs. 10(b) and 10(c). First, the acoustic energy density must be high enough for cells to ever reach their IAP, and this error depends on cell size, the starting position relative to the location of the IAP, and how fast the IAP moves spatially in the diffusing gradient. Large cells reach their IAP faster than small cells due to the strong size dependency [Eqs. 3(d) and 3(e)] and the determination will thus be more accurate for larger cells. Likewise, cells that start out closer to the spatial location of the IAP

TABLE II. Resonance frequencies from eigenfrequency simulations.

C_{ix}^{side} (%)	C_{ix}^{center} (%)	Z^{side} (MPa s/m)	Z^{center} (MPa s/m)	f_0 (MHz)	ΔC_{ix} (%)	Δf_0[a] (kHz)
10	10	1.58	1.58	2.001	0	0
20	20	1.66	1.66	1.994	0	–7
35	35	1.77	1.77	1.990	0	–11
20	35	1.66	1.77	1.961	15	–40
10	35	1.58	1.77	1.942	25	–59

[a] $\Delta f_0 = f_0 - 2.001$ MHz.

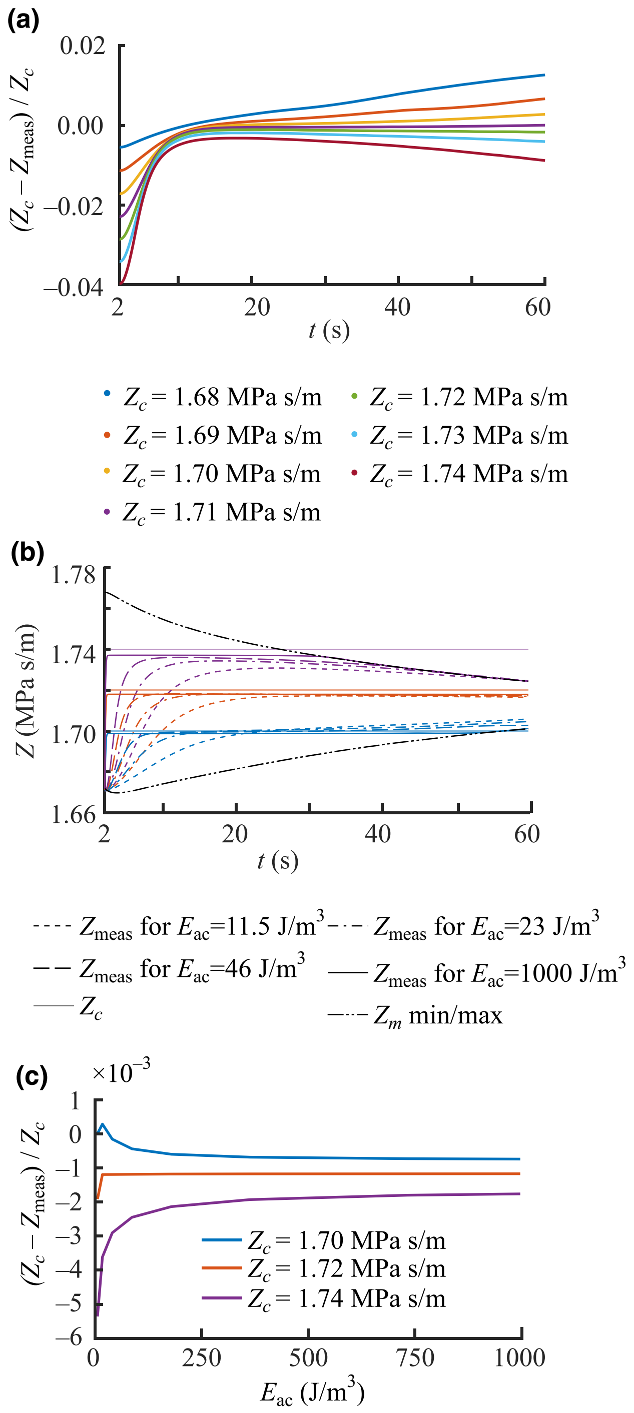

FIG. 10. Error when measuring the acoustic impedance of neutrophils. (a) Relative error when assigning $Z_c = Z_m$ for cells of $\rho_c = 1090$ kg/m^3 [56] for different Z_c. (b) Z_{meas} versus time for a range of Z_c and E_{ac}. (c) Average relative error in the time span $t = 20$ to 30 s for increasing E_{ac}. (b),(c) share the same color coding.

will also have smaller error due to a fast transition to the IAP. Since the spatial location of the IAP is constantly changing due to diffusion, cells will not be able to stay in their IAP over time if they are too small or if the acoustic energy density is too low. For the high acoustic energy, this error vanishes, and what remains is an approximation error that stems from the approximation that $\Phi = 0$ is equivalent to $Z_c = Z_m$. In Ref. [40], it is shown that for biological cells, this error will always be negative, and it can only be zero for combinations of ρ_c and κ_c that exactly match ρ_m and κ_m of the medium of solute molecules that forms the acoustic impedance gradient.

[1] C. J. O. Bacal, J. W. Maina, H. H. Nandurkar, M. Khaleel, R. Guijt, Y. Chang, K. M. Dwyer, and L. F. Dumée, Blood apheresis technologies – a critical review on challenges towards efficient blood separation and treatment, Mater. Adv. **2**, 7210 (2021).

[2] R. Nasiri, A. Shamloo, S. Ahadian, L. Amirifar, J. Akbari, M. J. Goudie, K. Lee, N. Ashammakhi, M. R. Dokmeci, D. Di Carlo, *et al.*, Microfluidic-based approaches in targeted cell/particle separation based on physical properties: Fundamentals and applications, Small **16**, 2000171 (2020).

[3] V. Laxmi, S. S. Joshi, and A. Agrawal, Extracting white blood cells from blood on microfluidics platform: A review of isolation techniques and working mechanisms, J. Micromech. Microeng. **32**, 053001 (2022).

[4] J. F. Edd, A. Mishra, K. C. Smith, R. Kapur, S. Maheswaran, D. A. Haber, and M. Toner, Isolation of circulating tumor cells, Iscience **25**, 104696 (2022).

[5] K. Abhishek, A. S. C. L. S. Titus, M. T. P. Dinh, A. Mukhamedshin, C. Mohan, S. C. Gifford, and S. S. Shevkoplyas, Red blood cell rosetting enables size-based separation of specific lymphocyte subsets from blood in a microfluidic device, Lab Chip **23**, 1804 (2023).

[6] N. Xiang, J. Wang, Q. Li, Y. Han, D. Huang, and Z. H. Ni, Precise size-based cell separation via the coupling of inertial microfluidics and deterministic lateral displacement, Anal. Chem. **91**, 10328 (2019).

[7] N. Norouzi, H. C. Bhakta, and W. H. Grover, Sorting cells by their density, PLoS One **12**, 0180520 (2017).

[8] M. Urbanska, H. E. Munoz, J. S. Bagnall, O. Otto, S. R. Manalis, D. Di Carlo, and J. Guck, A comparison of microfluidic methods for high-throughput cell deformability measurements, Nat. Methods **17**, 587 (2020).

[9] C. Honrado, P. Bisegna, N. S. Swami, and F. Caselli, Single-cell microfluidic impedance cytometry: From raw signals to cell phenotypes using data analytics, Lab Chip **21**, 22 (2021).

[10] K. Delikoyun, S. Yaman, E. Yilmaz, O. Sarigil, M. Anil-Inevi, K. Telli, O. Yalcin-Ozuysal, E. Ozcivici, and H. C. Tekin, Hologlev: A hybrid magnetic levitation platform integrated with lensless holographic microscopy for density-based cell analysis, ACS Sens. **6**, 2191 (2021).

[11] N. G. Durmus, C. Tekin, S. Guven, K. Sridhar, A. A. Yildiz, G. Calibasi, I. Ghiran, R. W. Davis, L. M. Steinmetz, and U. Demirci, Magnetic levitation of single cells, Proc. Natl. Acad. Sci. U.S.A. **112**, E3661 (2015).

[12] A. A. Nawaz, D. Soteriou, C. K. Xu, R. Goswami, M. Herbig, J. Guck, and S. Girardo, Image-based cell sorting using
